# Elocalcitol, a fluorinated vitamin D derivative, prevents high-fat diet-induced obesity via SCAP downregulation and miR-146a-associated mechanisms

**DOI:** 10.3389/fphar.2024.1505729

**Published:** 2025-01-17

**Authors:** Monika Jürgenson, Keerthana Chithanathan, Aivar Orav, Külli Jaako, Janeli Viil, Mithu Guha, Kalev Kask, Alexander Zharkovsky

**Affiliations:** ^1^ Department of Pharmacology, Institute of Biomedicine and Translational Medicine, University of Tartu, Tartu, Estonia; ^2^ Tartu University Hospital Joint Laboratory, Tartu University Hospital, Tartu, Estonia; ^3^ Adge Pharmaceuticals Inc., Mountain View, CA, United States

**Keywords:** elocalcitol, vitamin D derivative, high-fat diet (HFD), mouse model, obesity prevention, SCAP downregulation, SREBP-mediated lipogenesis, miR-146a

## Abstract

**Background:**

Obesity is an emerging health problem worldwide as it is associated with increased risk of cardiovascular, metabolic, mental disorders, and cancer. Therapeutic weight management remains one of the options for the treatment of excess weight and associated comorbidities. In this study, the therapeutic potential of elocalcitol, a fluorinated derivative of vitamin D, was studied on the model of high-fat diet (HFD)-induced obesity in mice.

**Results:**

It was demonstrated that co-administration of elocalcitol in the doses 15 ug/kg (i.p.) twice a week for 16 weeks prevented body weight gain by approximately 15%. The significant retardation in the body weight gain was observed already on the second week of elocalcitol treatment. Administration of elocalcitol also reduced visceral and epididymal fat accumulation by 55% and 35%, respectively, metabolic syndrome development, and lipid droplets accumulation in the liver of mice exposed to HFD. In contrast, the administration of cholecalciferol (vitamin D)—a precursor to calcitriol, the biologically active form of vitamin D, did not affect significantly the signs of obesity and metabolic syndrome, suggesting that the anti-obese effects of elocalcitol are not related to the canonical vitamin D receptor (VDR). Further studies have demonstrated that the preventive effect of elocalcitol is associated with the decreased levels of sterol regulatory element-binding protein (SREBP) cleavage-activating protein (SCAP) and upregulation insulin-inducing gene-1 (*Insig1*) mRNA expression suggesting that the anti-obese effect of elocalcitol is mediated via inhibition of SREBP-mediated lipogenesis. We also demonstrated that elocalcitol prevents an increase in the expression of proinflammatory cytokines such as interleukin-1 beta (*Il1b*), tumor necrosis factor-alpha (*Tnf*), and interleukin-18 (*Il18*), and this effect was associated with upregulation of microRNA-146a (miR-146a). Deletion of the miR-146a gene reduced the anti-obese effects of elocalcitol and prevented its actions on the SCAP levels. The data indicate that elocalcitol’s reduction of SCAP is at least partly mediated by miR-146a modulation.

**Conclusion:**

The study demonstrates that elocalcitol prevents HFD-induced obesity and metabolic syndrome in mice, likely by inhibiting SREBP-mediated lipogenesis and upregulating miR-146a. These findings provide valuable insights into the anti-obesity mechanisms of fluorinated D-vitamin analogs and suggest potential therapeutic strategies for obesity prevention.

## 1 Introduction

Obesity is defined as an excessive over-accumulation of body fat that adversely affects multiple aspects of health and represents a significant health problem worldwide (Afshin et al., 2017). Numerous data have demonstrated that vitamin D deficiency might be related to obesity and metabolic syndrome (Mansouri et al., 2018; Schmitt et al., 2018) and an inverse relationship between plasma vitamin D concentrations and the characteristics of obesity and metabolic syndrome (Melguizo-Rodríguez et al., 2021). Although obesity and metabolic syndrome are commonly associated with low vitamin D levels, the link between vitamin D deficiency and obesity remains controversial. This is because weight loss has minimal impact on improving vitamin D status, and vitamin D supplementation does not appear to promote weight loss (Karampela et al., 2021). Nevertheless, some studies have demonstrated that vitamin D decreased adipocyte lipid storage (Yin et al., 2012), reduced insulin resistance and hepatic steatosis and reduced obesity-induced inflammation in diet-induced obese mice (Marziou et al., 2020; Benetti et al., 2018). Furthermore, some prospective studies in humans point to a preventive effect of vitamin D supplementation on the onset of obesity (Bennour et al., 2022).

The primary supplemental form of vitamin D_3_ is also known as cholecalciferol. This form serves as a precursor to the biologically active form of vitamin D, which is 1,25-dihydroxyvitamin D_3_ (1α,25(OH)_2_D_3_) or calcitriol. Calcitriol mediates its biological effects by binding to and activating the vitamin D receptor, a member of the steroid hormone nuclear receptor family that functions as a transcription factor upon activation (Bikle, 2014).

However, calcitriol is also known to impact protein expression, oxidative stress, inflammation, and cellular metabolism through various genomic and non-genomic mechanisms (Hii and Ferrante, 2016). It should be noted that supplemental vitamin D_3_ exerts its immunomodulatory actions at supraphysiological concentrations, which, similarly to the administration of calcitriol, leads to hypercalcemia, hypercalciuria, and renal dysfunctions (Tebben et al., 2016). These observations have spawned a major effort to develop vitamin D analogs that can separate the effects on calcium homeostasis from the effects on other biological processes (Bikle, 2014). Today, more than 3,000 synthetic vitamin D analogs have been developed by various pharmaceutical companies and academic research groups with the main goal of identifying compounds with a lower calcemic effect (Maestro et al., 2019). With a very tight calcemic control, several parenterally administered synthetic vitamin D analogs have displayed a remarkable ability of a long-term weight control in normal mice (Smith et al., 2000). As traditional strategies to manage obesity, such as diet, exercise, or surgery, often yield limited long-term success (Hall and Kahan, 2018; Wadden et al., 2020), and pharmacological interventions, including GLP1 agonists and other synthetic drugs, can cause undesirable side effects (Coulter et al., 2018), there is a growing need for innovative pharmacological approaches.

Recently it was shown that 25-hydroxycholecalciferol, as well as certain fluorinated analogs of vitamin D, inhibit the activation of sterol regulatory element-binding proteins SREBP´s, a family of master transcription factors regulating lipogenesis and cholesterol metabolism (Kawagoe et al., 2021). Amongst them, SREBP-1c modulates the transcription of genes encoding enzymes necessary for lipogenesis such as acetyl CoA carboxylase, fatty acid synthase, stearoyl CoA desaturase-1, and lipoprotein lipase whereas SREBP-2 predominantly controls the expression of genes involved in cholesterogenesis (Brown and Goldstein, 1997; Shimano, 2001; Goldstein et al., 2006). Activation of transcriptional activity of SREBP´s starts from binding to SREBP cleavage-activating protein (SCAP), a specific escort protein for SREBP (Hua et al., 1996). SCAP plays an important role in the regulation of SREBP activity by transporting SREBP from the endoplasmatic reticulum to the Golgi apparatus, facilitating the release of the N-terminus of SREBP by site-1 and site-2 proteases and permitting its entry into the nucleus (DeBose-Boyd and Ye, 2018; Brown and Goldstein, 1997). SCAP also binds to endoplasmatic reticulum proteins, insulin-inducing gene-1 and 2 (Insig-1, Insig-2), and this complex induces retention of SCAP/SREBP complex in the endoplasmatic reticulum (ER) thereby preventing SREBP activation and consequent inhibition of its action on the lipogenesis gene transcription (Yang et al., 2002). A formed complex of 25-hydroxyvitamin D_3_ or certain other vitamin D analogs, but not cholecalciferol, trigger rapid SCAP degradation via ubiquitination thus reducing the availability of SCAP for SREBP (Asano et al., 2017; Kawagoe et al., 2021). The degradation of SCAP by vitamin D analogs occurs independently from the classical VDR and SCAP represents another genomic target for vitamin D analogs (Kawagoe et al., 2021; Kawagoe et al., 2023).

MicroRNAs (miRNAs) are small non-coding RNA molecules that regulate numerous biological processes, including the immune response. Emerging as one of the most important miRNAs to orchestrate immune and inflammatory signaling is microRNA-146a (miR-146a) (Taganov et al., 2006; Boldin et al., 2011). Recent studies have demonstrated the important roles of miR-146a in obesity and obesity-related comorbidities. It was shown that miR-146a reduced the inflammatory response in human adipocytes and, therefore, could contribute to the regulation of inflammatory processes in adipose tissue and possibly prevent an overwhelming inflammatory response (Roos et al., 2016; Roos et al., 2021). There are also data to suggest that miR-146a controls insulin sensitivity in adipocytes (Roos et al., 2021) as reduced miR-146a levels were associated with insulin resistance, poor glycemic control, and increased expression of several proinflammatory cytokine genes in Type 2 diabetic patients (Feng et al., 2018).

Elocalcitol or BXL-628 or Ro-26-9228 (1-α-fluoro-25-hydroxy-16,23E-diene-26,27-bishomo-20-epi-cholecalciferol) is a non-hypercalcemic 1-α-fluor-substituted calcitriol derivative (Peleg et al., 2002; Nagpal et al., 2005) shown to increase bone mineral density and decrease prostate growth in preclinical and clinical studies and demonstrates anti-proliferative and anti-inflammatory properties (Peleg et al., 2002; Adorini et al., 2007). The therapeutic potential of elocalcitol in obesity has not been explored, and, therefore, the present study aimed to investigate the effects of elocalcitol in comparison with vitamin D_3_ on the development of obesity and obesity-related metabolic syndrome in mice fed with a high-fat diet. We also aimed to explore in more detail whether SREBP-mediated lipogenesis and miR-146a-mediated inflammation are affected by elocalcitol’s treatment.

## 2 Materials and methods

### 2.1 Animals and experimental design

All experiments were conducted in compliance with the guidelines outlined in the *Principles of Laboratory Animal Care* (Directive 2010/63/EU). The experimental protocol was approved by the Animal Experimentation Committee of the Estonian Ministry of Agriculture (No. 177, 2020, and 1.2–17/166, 2023). Throughout the study, all mice were housed 5 animals per cage under controlled conditions, maintained on a 12:12-hour light/dark cycle at a temperature of 21°C–23°C and a relative humidity of 55% ± 10%, and had unrestricted access to food and water (*ad libitum*). At every stage of the experiment, all efforts were made to minimize the suffering of the animals.

Two distinct experiments were conducted: the first experiment aimed to assess the protective effects of elocalcitol compared to vitamin D_3_ (referred to as vitamin D) against obesity, and the second experiment to elucidate whether the potential mechanism of elocalcitol could be mediated via the miR146a expression pattern. The first experiment lasted for 16 weeks. Sixty male C57BL/6J mice, aged 6 weeks and with an average body weight of 22 g, were obtained from Envigo RMS B.V. (Horst, Netherlands) and divided into four experimental groups (N = 15, per group) receiving either a low-fat diet or a high-fat-diet providing either 10% (3.61 kcal/kg) or 45% (4.65 kcal/kg) of total energy as fat (low-fat diet consisted 10% fat as lard, 20% protein, and 70% carbohydrates (7% sugar) while high-fat diet consisted 45% fat as lard, 20% protein and 35% carbohydrates (21% sugar); E157453-04, E15744-344, ssniff Spezialdiäten GmbH, Germany): (1) mice fed with low-fat diet (referred to as Control), (2) mice fed a high-fat diet (referred to as HFD), (3) mice fed a high-fat diet and treated with vitamin D (referred to as HFD + VitD), and (4) mice fed a high-fat diet and treated with elocalcitol (referred to as HFD + Eloc). Vitamin D (cholecalciferol, C9756 (purity ≥ 98%), Sigma-Aldrich, Germany, diluted in ethanol) and elocalcitol (SML23-95 (purity ≥ 98%), Sigma-Aldrich, Germany, diluted in ethanol) were administered intraperitoneally at a dosage of 15 μg/kg twice a week for 16 weeks, while the control and high-fat diet groups received a vehicle (0.9% saline containing 0.3% ethanol) solution.

The second experiment lasted for 8 weeks. Forty six-week-old male miR-146a knockout (KO) mice and 40 corresponding wild-type (WT) mice were acquired by crossing miR-146a ± heterozygous mice bred and maintained following the regulations of the Laboratory Animal Centre at the Institute of Biomedicine and Translational Medicine, University of Tartu. The animals produced were genotyped using the following primer sequences:

146a locus 5′forward primer- 5′- ACC AGC AGT CCT CTT GAT GC-3′,
146a locus 3′reverse primer- 5′GAC GAG CTG CTT CAA GTT CC-3′

and same-generation littermates were used in experiments. Both, WT and KO animals were divided into 4 experimental groups (N = 10, per group) receiving (1) LFD + vehicle (referred to as WT Control/KO Control), (2) HFD + vehicle (referred to as WT HFD/KO HFD), (3) LFD + elocalcitol (referred to as WT + Eloc/KO + Eloc) (4) HFD + elocalcitol (referred to as WT HFD + Eloc/KO HFD + Eloc). Elocalcitol (30 μg/kg) was administrated intraperitoneally twice a week lasting for 8 weeks while WT Control/KO Control and WT HFD/KO HFD groups were administrated vehicle solution.

The dosages of elocalcitol used in these experiments (15 μg/kg and 30 μg/kg, i. p., twice weekly) were selected based on prior dose-response studies, which indicated that significant therapeutic effects could be achieved with doses well below the maximum tolerated dose, particularly concerning the risk of hypercalcemia. For example, a dose of 3 μg/kg resulted in a 60% reduction in intraprostatic infiltrates, demonstrating strong efficacy at a safe level (Penna et al., 2006). Additionally, pharmacokinetic studies in rats (Peleg et al., 2002) indicated that parenteral administration of elocalcitol results in a longer half-life compared to oral administration, with sufficient area under the curve (AUC) to support a twice-weekly injection regimen, as opposed to the thrice-weekly dosing used in previous studies (Smith et al., 2000).

### 2.2 Body weight and food and water consumption

Animals’ body weights, were monitored on a weekly basis throughout the study. The 24-hour food and water intake were measured once a month.

### 2.3 Glucose tolerance test

For glucose tolerance test (GTT) mice were fasted overnight for 10–12 h and blood glucose was measured from the tail blood using Accu-Chek Performa system glucose meter (Accu-Chek^®^ Performa, Roche, Mannheim, Germany) and the baseline glucose values (0 min) were recorded. The animals were then injected intraperitoneally with glucose (2 g/kg) and after glucose injection the blood glucose was measured from tail blood at regular intervals: 15, 30, 60, 90, and 120 min.

### 2.4 Insulin tolerance test

For the insulin tolerance test (ITT), mice were fasted for 6 h. Blood glucose levels were measured from tail blood using the Accu-Chek Performa glucose meter (Accu-Chek^®^ Performa, Roche, Mannheim, Germany), and baseline glucose values at 0 min (min) were recorded. The animals were then injected intraperitoneally with insulin 0.5 U/kg; diluted from 100 U/mL (Novorapid, Novo Nordisk A/S) with 0.9% saline and after glucose injection (2 g/kg), the blood glucose was measured from tail blood at regular intervals: 15, 30, 60, 90, and 120 min.

### 2.5 *In vivo* magnetic resonance imaging

Body composition analysis for the first part of the experiment was conducted on anesthetized mice using a magnetic resonance imaging (MRI) system (Bruker BioSpin Group, Bruker Corporation, Germany). The abdominal area of animals was scanned for assessing visceral and subcutaneous fat loading before the experiment and at the end of the experiment. The procedure was conducted as described previously (Plaas et al., 2017). In summary: mice (n = 5 per group) were anaesthetized using isoflurane (1.5%–2.5% in 1.5 L/min medical oxygen) and placed on a heated animal bed throughout the MRI procedure. Scans were performed using a 9.4T Bruker BioSpec 94/21 USR system connected to a 1 H circular polarized transceiver coil and running ParaVision 6.0.1^®^ software (Bruker BioSpin Group, Bruker Corporations, Germany). Respiration was monitored using a respiration pillow (SA Instruments Inc., Stony Brook, United States) and respiration rate was maintained at between 35 and 70 breaths per min. Two orientation pilot scans were performed to establish the animal’s position and identify anatomical landmarks relevant for planning the subsequent scan. The final T1-weighted Bruker:RARE sequence was performed using the following parameters: repetition time (TR) 1,164 m, echo time (TE) 6 m, flip angle 90°, number of averages 2, imaging matrix 320 × 320 × 40, spatial resolution 0.125 × 0.125 × 0.5 mm; number of scanned images per animal: n = 41. Volumes were segmented manually by an observer blinded to the experiment using ITK-SNAP (V3.8.0). A × 40 magnification was employed to assess the volumes in cubic millimeters (mm³) of visceral and subcutaneous fat, with the renal region serving as the reference point for fat volume analysis. The results were reported as a percentage of total body volume for fat and in mm³ for spinal muscles.

### 2.6 Blood samples and organs

Twenty-four hours after the last administration, the animals were deeply anesthetized with a terminal dose of pentobarbital sodium (200–300 mg/kg). Blood samples were collected from all groups via cardiac puncture, following a standardized protocol. The collected blood was allowed to coagulate and was then centrifuged at 2000 g for 20 min. The serum was stored at −80°C until analysis. To assess the effects of a high-fat diet on animal organs, the epididymal adipose tissue and liver were excised, weighed, and subsequently stored at −80°C for further processing. Urine samples were collected from the mice during the final week of the experiment. To obtain the samples, gentle pressure was applied to the lower abdomen (above the bladder) while stimulating urination by rubbing towards the urethral meatus. The urine was then collected using a pipette and stored at −80°C until further analysis.

### 2.7 Biochemical analysis of the serum and urine

The biochemical measurement of calcium concentration in serum and urine was performed in the Tartu Healthcare College by using the Calcium ArsenazoIII Colorimetric Method (Giesse Diagnostics, Italy) according to standard operating procedures.

### 2.8 Oil Red O staining

To evaluate lipid accumulation in the liver, Oil Red O staining was conducted to identify and quantify lipid droplets, providing insights into fat storage within tissues (Demaret et al., 2020). Animals were deeply anesthetized with a terminal dose of pentobarbital sodium (300 mg/kg, i. p.) and subsequently transcardially perfused first with 0.9% saline, followed by 4% phosphate-buffered paraformaldehyde (ROTI®Histofix, Carl Roth GmbH, Germany). The fixed livers were maintained in the 4% paraformaldehyde solution until processing. To evaluate lipid accumulation in the liver, Oil Red O staining was performed. Livers were infiltrated with 30% sucrose for 4 days, then embedded in tissue freezing media (Leica Biosystems, IL, United States). The frozen blocks were stored overnight at −30°C and then sectioned at 10 μm using a cryostat (CM 1860, Leica Biosystems, IL, United States). For staining, the slides were incubated in the Oil Red O working solution (O0625, Sigma-Aldrich, Germany) for 15 min. After being washed with water the sections were dried and cover-slipped with aqueous glycerine-glycerol mounting medium. Microscope images (3 images per section, 4 sections per animal, n = 5) were captured by using a Leica ICC50HD camera and DM500 microscope with ×20 magnification. For quantification of Oil Red O positive signal in stained liver sections image analysis software was used (ImageJ 1.48v, http://imagej.nih.gov/ij). Briefly, images were converted to an 8-bit format, and the background was subtracted. Next, images were thresholded using adjusted threshold command ensuring that all areas of Oil Red O appeared to be included. Finally, the image layer was binarized and % area covered by Oil Red O staining was measured.

### 2.9 Total RNA isolation and real-time quantitative PCR

Total RNAs were extracted from epididymal fat and liver tissues. For epididymal fat RNA was prepared using RNeasy lipid tissue mini kit (74,804, Qiagen, Germany), following the instruction manual and liver RNA was extracted with TRI Reagent^®^ (TR 118) (Molecular Research Center, Inc., Cincinnati, OH, United States). RNA quantity and purity were measured by NanoDrop (Thermo Fisher Scientific). To measure mRNA expression, cDNA was synthesized using RevertAid First Strand cDNA Synthesis Kit with Oligo (dT) primers (Thermo Fisher Scientific, United States) followed by qPCR using 5 × HOT FIREPol^®^ EvaGreen^®^ qPCR Supermix (Solis BioDyne, Tartu, Estonia) on a QuantStudio 12KFlex system (Thermo Fisher Scientific). Primer sequences for target genes are given in Supplementary Table S1. For qPCR analysis, triplicates of each sample were used, and the average Ct value was calculated. The Ct values of the target gene were normalized to the housekeeping gene Gapdh for mRNA quantification and U6 for miRNA analysis, to obtain the ΔCt values. The ΔCt of each sample was then compared to the ΔCt of a calibrator sample (control) to calculate ΔΔCt. Relative expression levels were determined using the formula 2^−ΔΔCT^, where the control sample was normalized to 1, and other samples were expressed relative to the control (Livak and Schmittgen, 2001).

### 2.10 Western blot analysis

Liver tissues (15–20 mg) were lysed in lysis buffer (20 mM Tris-HCl, pH 7.5, containing protease inhibitors) for protein extraction. The protein concentration was determined using the Bradford reagent kit (B6916, Sigma-Aldrich, Germany). For Western blot analysis, 20 µg of total protein for SCAP detection was dissolved in 8% sodium dodecyl sulfate-polyacrylamide gels and transferred electrophoretically onto an Immobilon-FL PVDF membrane (Merck Millipore, Germany). The membranes were blocked with Intercept (TBS) Blocking Buffer (Li-Cor Biosciences, United States) for 60min at room temperature. Then, the membranes were incubated for 48 h at 4°C with the primary antibody for SCAP (Rb, ab308060, 1:1,000, Abcam; United Kingdom) and after washing thrice with TBST (50 mmol/L Tris, pH 7.6; 0.9% NaCl; and 0.1% Tween-20), the membranes were incubated with the secondary antibody (IRdye800, aRb, 1:10,000, 926–32211, Li-Cor Biosciences, United States) for 1 h at RT. Housekeeping protein GAPDH as protein loading control was detected by incubating membranes overnight with monoclonal anti-GAPDH antibody (Ms, ab8245, 1:10,000, Abcam, United Kingdom), followed by incubation with IRDye conjugated secondary antibody (IRdye680LT, aMs, 1:10,000, 926–68020, Li-Cor Biosciences, United States). After extensive washing with TBST, the Odyssey Infrared Imaging System (Li-Cor Biosciences, United States) was used for the detection and quantification. The ratio of proteins of interest to GAPDH was calculated as the mean OD ratio in arbitrary units and presented as % of change as compared to the control vehicle group ±standard error of mean (SEM).

### 2.11 Statistical analysis

GraphPad 9.4.1 (San Diego, CA, US) was used for statistical analyses and graphical presentations. Data sets were tested for homogeneity of variances using the Brown-Forsythe test and for normal distribution using the Shapiro-Wilk test. One-way ANOVA or two-way ANOVA followed by multiple comparisons Tukey´s *post hoc* test, was used, and statistical significance was set at p < 0.05. In all figures, data are shown as mean ± standard error of the mean (SEM).

## 3 Results

### 3.1 Effects of vitamin D and elocalcitol on body weight and fat storage

**FIGURE 1:**
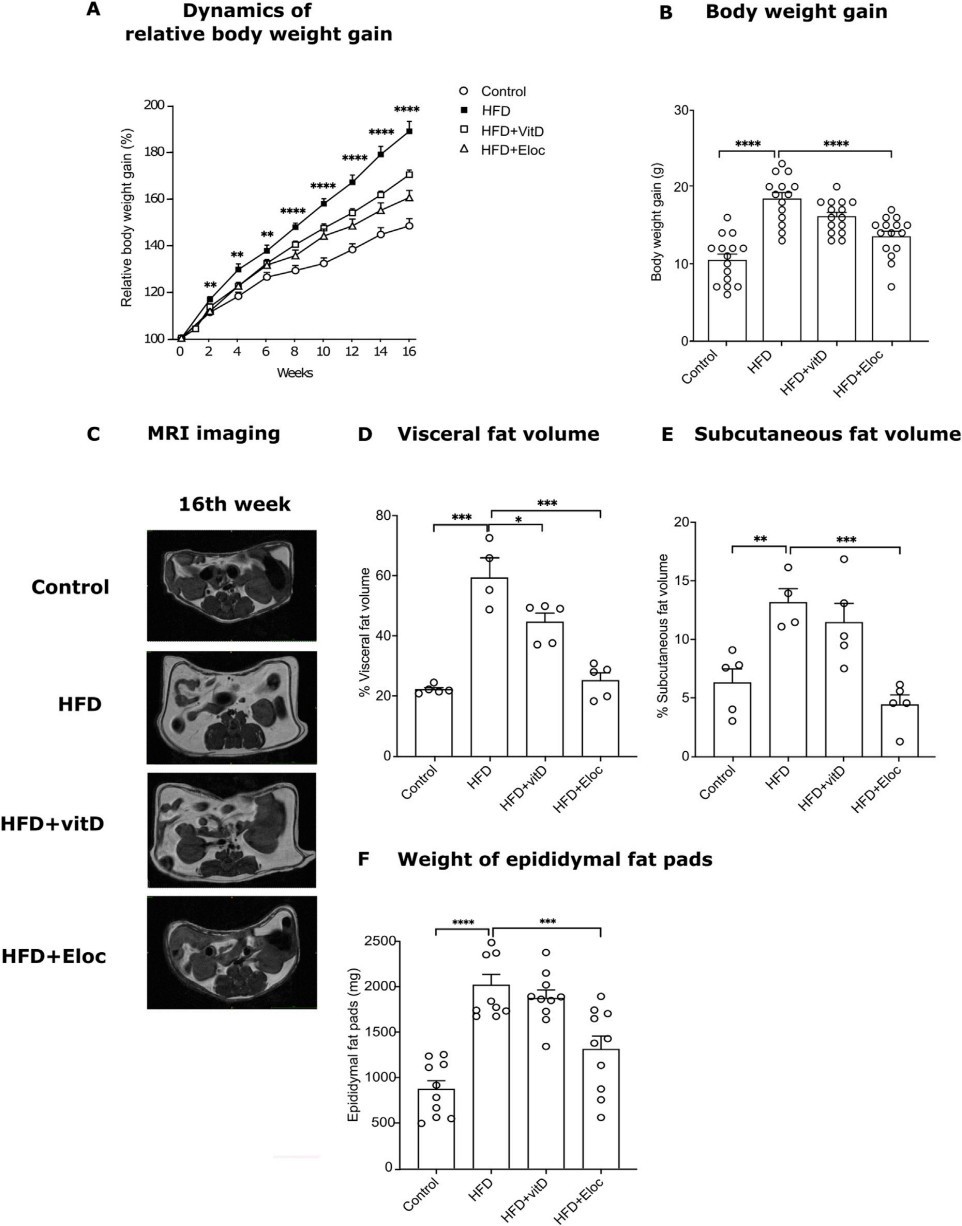
The effect of vitamin D and elocalcitol on the prevention of body weight gain and accumulation of fat storage in mice fed with HFD. **(A)** Dynamics of relative body weight gain; **p < 0.01; ****p < 0.0001; Two-way ANOVA followed by Tukey’s multiple comparisons *post hoc* test; **(B)** Body weight gain by the 16th week of treatment; **(C)** Representative MRI scans; **(D)** Visceral fat volume (%); **(E)** Subcutaneous fat volume (%); **(F)** Weight of epididymal fat pads (g). *p < 0.05; **p < 0.01; ***p < 0.001; ****p < 0.0001; One-way ANOVA followed by Tukey’s multiple comparisons *post hoc* test. Number of animals A–B: n = 15; D–E: n = 4–5; F: n = 10. The data are expressed as mean ± SEM. Abbreviations: Control, low fat diet; HFD, high-fat diet; HFD + VitD, high-fat diet treated with vitamin D; HFD + Eloc, high-fat diet treated with elocalcitol; MRI, magnet resonance imaging.

HFD induced significantly higher body weight gain as compared to control mice during 16 weeks of feeding (Figures 1A, B). Statistically significant effect (p < 0.01) of HFD on the body weight was observed already on the second week of HFD supplementation and continued to increase until the end of the experiment (16th week, p < 0.0001). Repeated administration of elocalcitol prevented HFD-induced body weight gain. The statistically significant effect (p < 0.01) of elocalcitol became evident on week eighth (Supplementary Figure S3) and became maximal at the end of the experiment (p < 0.0001, Figure 1B). Vitamin D_3_ also retarded the body weight gain of HFD-fed mice (Figure 1A), but its effect was considerably weaker than those observed in elocalcitol group. At the end of the experiment (16th week of treatment), the effect of vitamin D_3_ failed to reach the level of significance (p = 0.054; Figure 1B). Neither elocalcitol nor vitamin D_3_ induced hypercalcemia or calciuria (Supplementary Table S2). Monthly food and water consumption measurements did not reveal any significant differences between groups throughout the experiment (Supplementary Figure S1). However, considering the significantly higher physiological fuel value of the HFD feed (4.615 kcal/kg in HFD group vs. 3.630 kcal/kg in the control group), groups fed with HFD received a greater amount of metabolizable energy compared to the control group.

**FIGURE 2:**
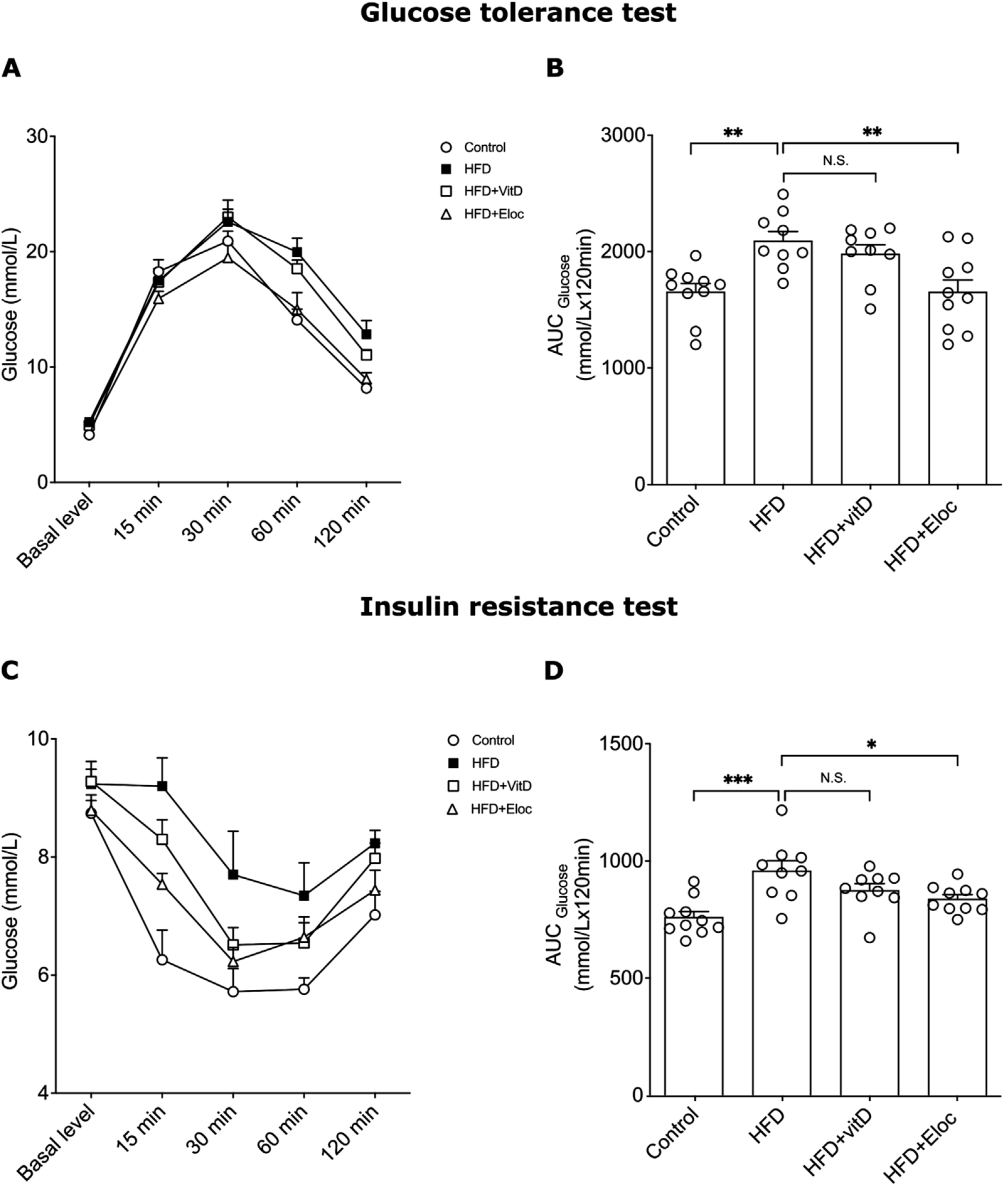
The effect of vitamin D and elocalcitol on the development of glucose homeostasis and insulin resistance in HFD-fed mice. **(A, B)** Glucose tolerance test; **(C, D)** Insulin resistance test. *p < 0.05; **p < 0.01; ***p < 0.001; One-way ANOVA followed by Tukey’s multiple comparisons *post hoc* test. Number of animals n = 9–10. The data are expressed as mean ± SEM. Abbreviations: Control, low fat diet; HFD, high-fat diet; HFD + VitD, high-fat diet treated with vitamin D; HFD + Eloc, high-fat diet treated with elocalcitol; AUC, area under curve.

An MRI-assisted analysis (Figure 1C) was used to quantify the visceral and subcutaneous fat as well as spinal muscles in the abdominal cavity of the experimental animals. In addition, the epididymal fat pads were weighed. HFD induced a significant increase in both visceral (p < 0.001, Figure 1D) and subcutaneous fat volume (p < 0.01, Figure 1E) by the 16th week of treatment. Similarly, the weight of the epididymal fat pad was significantly (p < 0.0001) higher in HFD compared to control mice (Figure 2F). Vitamin D significantly retarded the accumulation of visceral fat in HFD mice (p < 0.05, Figure 1D). The volume of subcutaneous fat as well as the weight of epididymal fat were more resistant to the effects of vitamin D and did not significantly change by its treatment (Figures 1E, F). Elocalcitol significantly retarded fat accumulation in all fat compartments: visceral fat (p < 0.001), subcutaneous fat (p < 0.001), and epididymal fat (p < 0.001; Figures 1D–F). Our results also indicate that neither HFD nor treatments influenced spinal muscle volume (Supplementary Figure S2). Neither HFD nor treatment influenced spinal muscle volume, and food intake remained consistent across groups despite differences in metabolizable energy (Supplementary Figures S1, S2).

### 3.2 The effect of vitamin D and elocalcitol on hyperglycemia in glucose tolerance test and insulin resistance in animals subjected to a high-fat diet

To assess the potential beneficial effects of vitamin D and elocalcitol on the prevention of hyperglycemia and insulin sensitivity in HFD-fed mice, the glucose tolerance test (GTT) and insulin tolerance test (ITT) were performed during the final 16th week of experiments. HFD induced a significant glucose intolerance following intraperitoneal glucose administration (2 g/kg) when compared with controls, as could be seen on the representative time point curve and determined by glucose area under the curve (AUC) (Figures 2A, B). Administration of vitamin D did not affect glucose AUC in HFD animals, while administration of elocalcitol, resulted in significantly lower glucose AUC compared to obese HFD mice (one-way ANOVA, Tukey’s *post hoc* test; p < 0.01; Figure 2B). HFD also induced an increased insulin resistance (p < 0.001; Figures 2C, D). Elocalcitol demonstrated a clear protective effect on insulin sensitivity which remained on the levels of control (p < 0.05; Figure 2D). By contrast, treatment with vitamin D did not affect insulin resistance of HFD-fed mice (Figure 2D). Thus, elocalcitol significantly prevented the development of glucose tolerance and insulin sensitivity in HFD-fed mice, reducing glucose AUC and maintaining insulin sensitivity at levels comparable to control mice. In contrast, vitamin D had no significant impact on either glucose tolerance or insulin resistance in these animals.

### 3.3 The effect of vitamin D and elocalcitol on pro- and anti-inflammatory cytokines in fat tissue of animals subjected to a high-fat diet

**FIGURE 3:**
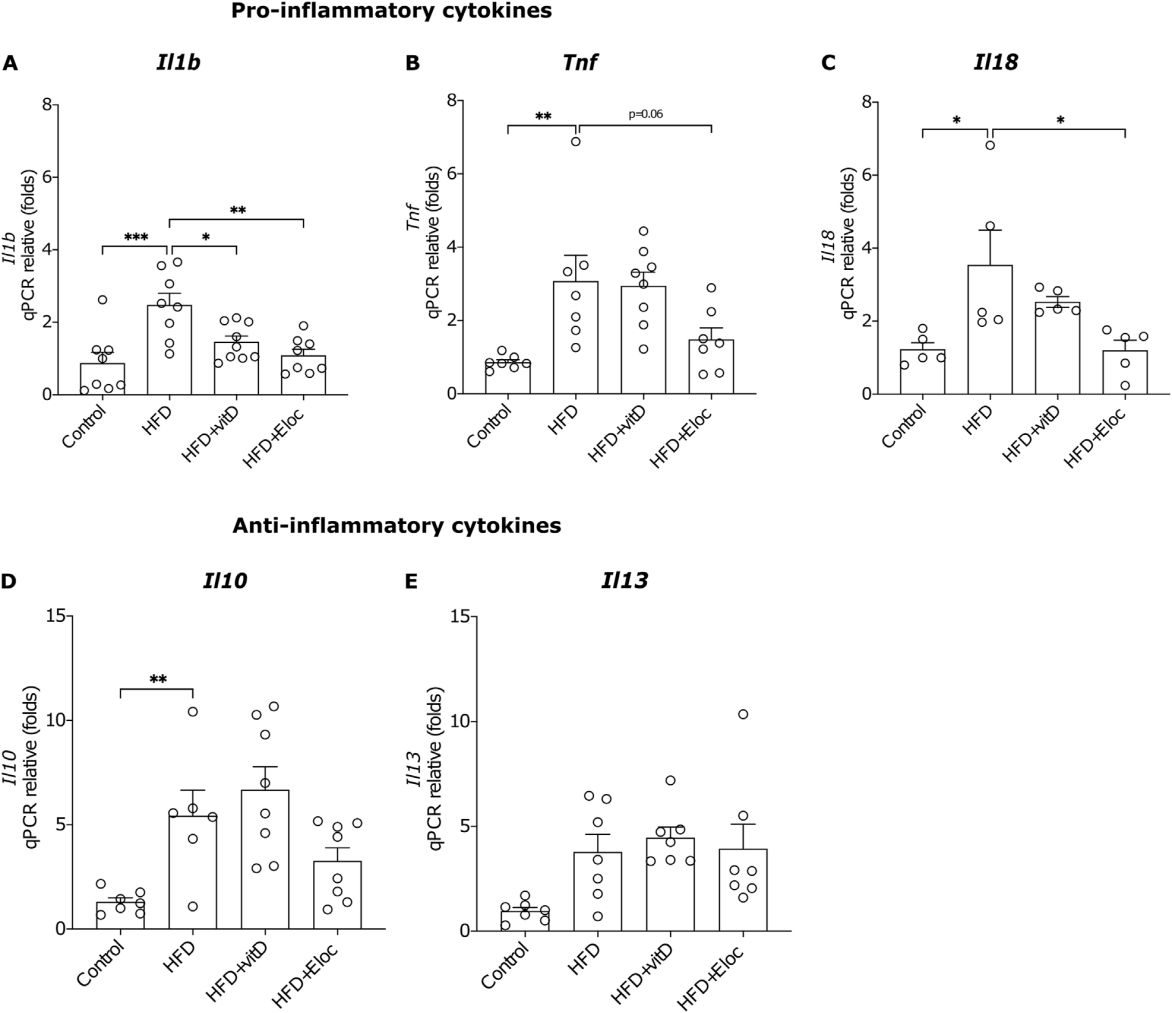
The effect of vitamin D and elocalcitol on the expression of pro- and anti-inflammatory cytokines in the epididymal fat tissue of obese mice. **(A–C)** Pro-inflammatory cytokines; **(D, E)** Anti-inflammatory cytokines. *p < 0.05; **p < 0.01; ***p < 0.001; ****p < 0.0001; One-way ANOVA followed by Tukey’s multiple comparisons *post hoc* test. Number of animals A–B: n = 7–8; C: n = 5; D–E: n = 7–8. The data are expressed as mean ± SEM. Abbreviations: Control, low-fat diet; HFD, high-fat diet; HFD + VitD, high-fat diet treated with vitamin D; HFD + Eloc, high-fat diet treated with elocalcitol; *Il1β*, interleukin-1 beta; *Tnf*, tumor necrosis factor-alpha; *Il18*, interleukin-18; *Il10*, interleukin-10; *Il13*, interleukin-13.

Numerous data suggest that the effect of vitamin D is attributed to its anti-inflammatory and immunomodulatory actions (Karkeni et al., 2018; Narvaez et al., 2009; Farhangi et al., 2017; Ding et al., 2016), and, therefore, we studied in more detail the effects of vitamin D and elocalcitol on the expression of pro- and anti-inflammatory cytokines. HFD significantly upregulated the mRNA expression of pro-inflammatory cytokines *Il1ß*, *Tnf*, and *Il18* (p < 0.001; p < 0.01; p < 0.05, respectively; Figures 3A–C). Elocalcitol successfully prevented the increase in all three pro-inflammatory markers, whereas vitamin D only prevented the rise of *Il1ß (*p < 0.05; Figure 3A), failing to affect *Tnf* and *Il18* expression. Additionally, HFD elevated the expression of the anti-inflammatory cytokine *Il10* (p < 0.01; Figure 3D); however, neither vitamin D nor elocalcitol influenced the expression of anti-inflammatory cytokines. These results suggest that elocalcitol exerts stronger anti-inflammatory effects compared to vitamin D in HFD-fed mice.

### 3.4 The effect of vitamin D and elocalcitol on hepatic steatosis and *Srebp1*-mediated lipogenesis

**FIGURE 4:**
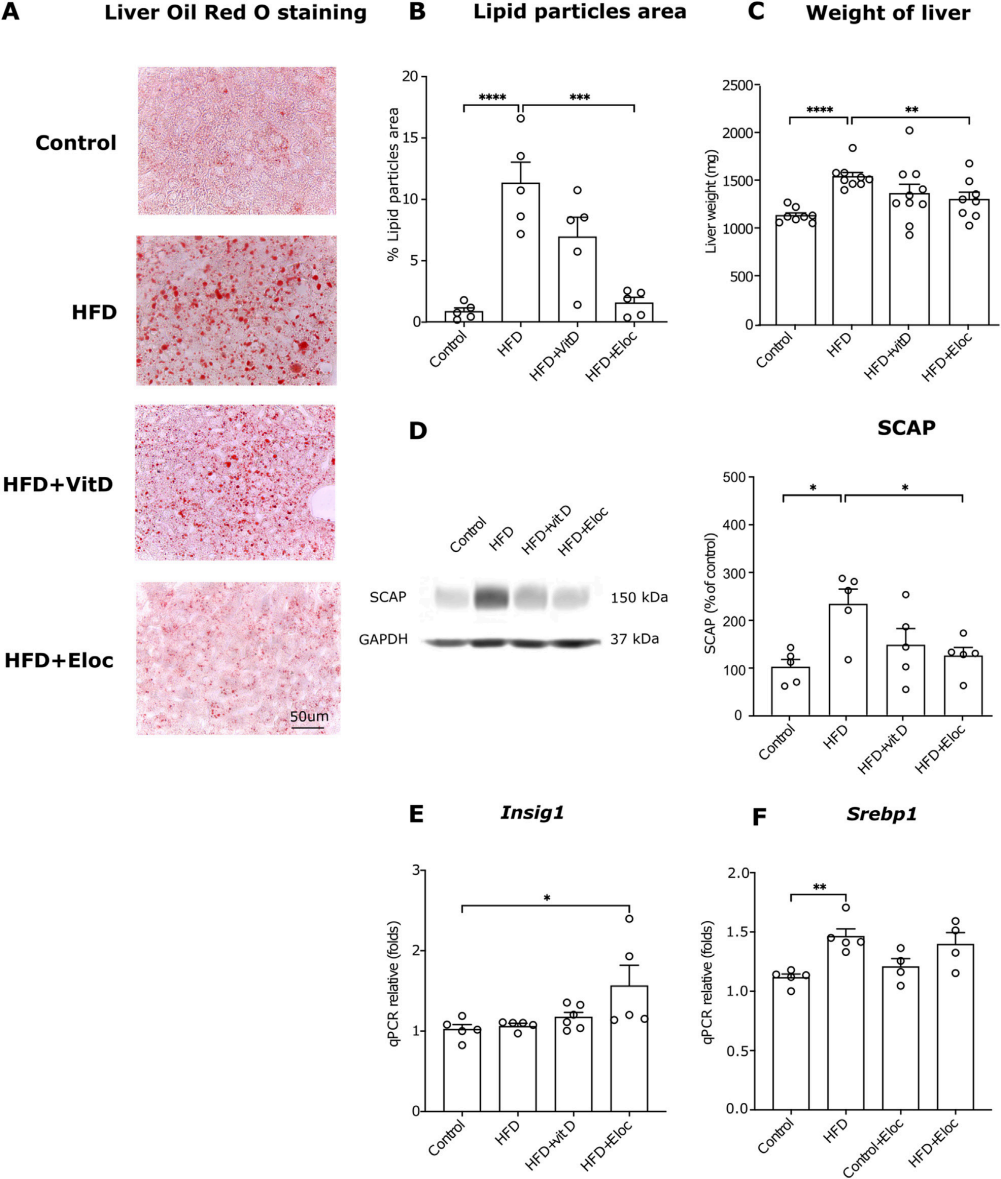
The effects of elocalcitol and vitamin D on hepatic steatosis and SREBP-mediated lipogenesis. **(A)** Representative microphotographs of lipid droplets; **(B)** Image analysis data of the effects of elocalcitol and vitamin D on the lipid particles area in liver sections of HFD mice; **(C)** Effect of elocalcitol and vitamin D on the weight of liver in HFD mice; **(D)** Effect of elocalcitol and vitamin D on the SCAP protein levels in liver tissue of HFD mice; **(E)** Effect of elocalcitol and vitamin D on the expression of *Insig1* mRNA in the liver of HFD mice; **(F)** Effect of elocalcitol and vitamin D on the expression of *Srebp1* mRNA in the liver of HFD mice. *p < 0.05; **p < 0.01; ***p < 0.001; ****p < 0.0001; One-way ANOVA followed by Tukey’s multiple comparisons *post hoc* test. Number of animals B: n = 5; C: n = 8–10; D–F: n = 4–6. The data are expressed as mean ± SEM. Abbreviations: Control, low-fat diet; HFD, high-fat diet; HFD + VitD, high-fat diet treated with vitamin D; HFD + Eloc, high-fat diet treated with elocalcitol; Srebp1, sterol regulatory element-binding protein 1c; SCAP, sterol regulatory element-binding protein cleavage-activating protein; Insig1, insulin-induced gene 1; Gapdh, glyceraldehyde-3-phosphate dehydrogenase.

In accordance with previous studies HFD treatment significantly induced accumulation of lipid droplets in hepatocytes (p < 0.0001; Figures 4A, B). In the HFD groups, treated with elocalcitol or vitamin D, fewer lipid droplets were observed in the liver as compared to the HFD group. Statistical analysis revealed a relevant decrease in the % of the area covered by Oil Red O positive signal in the livers of animals treated with elocalcitol (p < 0.001) but not with vitamin D (Figure 4B). In addition, the liver weights were also reduced in the HFD group that received elocalcitol (p < 0.01; Figure 4C). Next, we aimed to elucidate whether the observed preventive action of elocalcitol on HFD-induced obesity is linked to the suppression of lipogenesis mediated via the SERBP-1 pathway. We measured the levels of SCAP, an essential protein for SREBP processing. SCAP forms complexes with SREBP and escorts SREBP to Golgi for proteolytic processing, resulting in the cleavage of transcriptionally active SREBP (DeBose-Boyd and Ye, 2018). By measuring SCAP in the lysates of the liver tissue using Western blotting, we found that exposure to HFD increased SCAP protein levels (p < 0.05) while elocalcitol significantly (p < 0.05) prevented this increase (Figure 4D). Vitamin D also tended to decrease SCAP protein levels, but the effect did not reach a statistically significant difference. We also measured the expression of *Insig1* mRNA using qPCR and found a significant increase in HFD + Elocalcitol group compared to the control group (p < 0.05; Figure 4E). Although HFD induced an increase in expression of *Srebp1* (p < 0.05), elocalcitol did not affect the HFD-induced expression of *Srebp1* mRNA (Figure 4F).

### 3.5 The roles of miR-146a in the effects of vitamin D and elocalcitol on the development of obesity and metabolic syndrome in animals subjected to a high-fat diet

**FIGURE 5:**
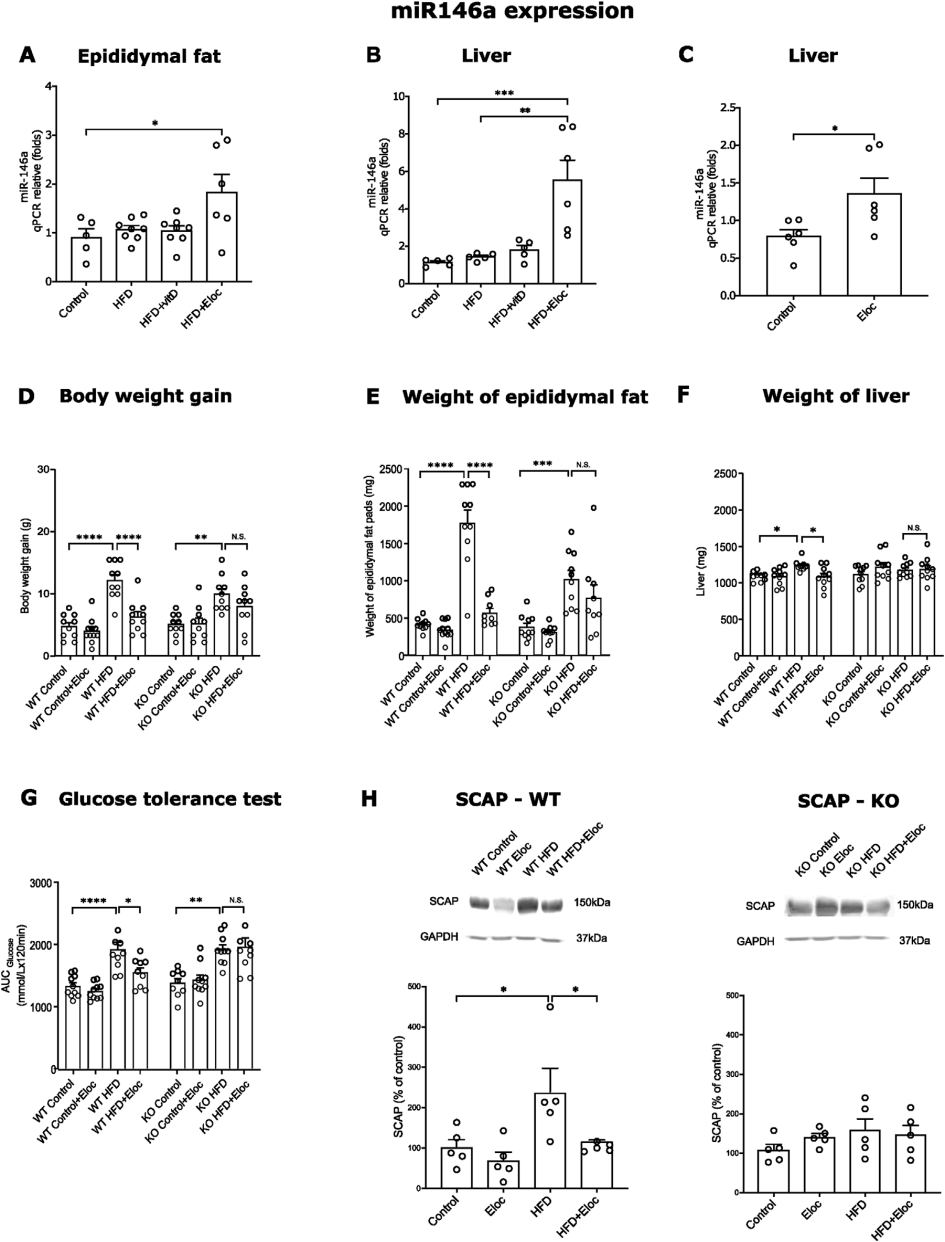
miR-146a expression in HFD fed mice and impact of miR-146a deficiency on the prevention of obesity by elocalcitol. **(A–C)** Expression of miR-146a in epididymal fat and liver; **(D–G)** The effects elocalcitol on the body weight gain, weight of epididymal fat pads, liver weight, and glucose tolerance in wild-type (WT) and miR-146a knockout (KO) subgroups fed with HFD; **(H)** The effect of elocalcitol on SCAP protein levels in WT mice and miR-146a KO mice. *p < 0.05; **p < 0.01; ***p < 0.001 One-way ANOVA followed by Tukey’s multiple comparisons *post hoc* test. Number of animals A: n = 7–8; B, C: n = 5–6; D: n = 10; E, F, G: n = 8–9; H: n = 5. The data are expressed as mean ± SEM. Abbreviations: Control, low-fat diet; HFD, high-fat diet; HFD + VitD, high-fat diet treated with vitamin D; HFD + Eloc, high-fat diet treated with elocalcitol; WT, wild-type animals; KO, miR-146a deficient animals; WT Control/KO Control, low-fat diet; WT HFD/KO HFD, high-fat diet; WT + Eloc/KO + Eloc, low-fat diet treated with elocalcitol; WT HFD + Eloc/KO HFD + Eloc, high-fat diet treated with elocalcitol; AUC, area under curve; SCAP, sterol regulatory element-binding protein cleavage-activating protein; Insig1, insulin-induced gene 1; Gapdh, glyceraldehyde-3-phosphate dehydrogenase.

Previous studies have demonstrated that the miR-146a expression is negatively correlated with insulin resistance, poor glycemic control, and the expression of pro-inflammatory cytokines such as Tnfα. Additionally, miR-146a has been found to inhibit lipogenesis induced by Tnfα (Wu et al., 2016). We, therefore, investigated the expression of miR-146a in the HFD induction of obesity and the effects of vitamin D and elocalcitol. In our experiments, HFD did not affect the expression levels of miR-146a in epididymal fat and liver tissues. However, treatment with elocalcitol resulted in a significant upregulation of miR-146a in epididymal fat (p < 0.05; Figure 5A) when compared to control mice. Additionally, elocalcitol treatment also induced significant increases in miR-146a expression in liver tissue for both obese and lean animals (p < 0.01 and p < 0.05, respectively; Figures 5B, C). This observed upregulation of miR-146a by elocalcitol in obese mice prompted us to explore the functional significance of this upregulation in the development of obesity and metabolic syndrome in more detail using constitutive miR-146a knockout mice and their WT littermates. Considering that the first experiment showed a significant weight gain in the HFD group within just 8 weeks (Supplementary Figure S3), we decided to shorten the duration of this HFD treatment by half. Additionally, in these experiments, elocalcitol was administered at a higher dose of 30 μg/kg (i.p.) twice a week, with no observed signs of hypercalcemia by the end of the study (Supplementary Table S3). Treatment with HFD resulted in a similar increase in body weight gain and epididymal fat accumulation in both WT and miR-146a KO mice (Figure 5D). However, in WT mice, treatment with elocalcitol significantly reduced HFD-induced weight gain (p < 0.0001; Figure 5D) and fat accumulation (p < 0.0001; Figure 5E), an effect that was not observed in miR-146a KO mice (Figures 5D, E). Additionally, HFD treatment in WT mice led to an increase in liver weight (p < 0.05; Figure 5F) and impaired glucose tolerance (p < 0.0001; Figure 5G), while miR-146a KO mice exhibited no significant changes in liver weight, though there was a significantly altered glucose tolerance (p < 0.01; Figure 5G). Elocalcitol treatment in WT mice further reduced liver weight and improved glucose tolerance, but these effects were absent in miR-146a KO mice (Figures 5F, G). Notably, when elocalcitol was administered to WT or miR-146a KO lean mice, there were no significant changes observed in body weight, epididymal fat accumulation, liver weight, or glucose tolerance (Figures 5D–G).

These data highlight the critical role of miR-146a in the protective effects of elocalcitol against the development of obesity and metabolic syndrome. To further investigate the role of miR-146a in SREBP-mediated lipogenesis, we measured SCAP protein levels in HFD-exposed WT and miR-146a knockout mice. In WT mice, HFD exposure led to elevated SCAP levels, which were significantly reduced upon treatment with elocalcitol (p < 0.05; Figure 5H). In contrast, miR-146a KO mice showed no changes in SCAP levels following either HFD exposure or elocalcitol treatment (Figure 5H). In summary, these results highlight the essential role of miR-146a in mediating the protective effects of elocalcitol against HFD-induced obesity and metabolic syndrome. Furthermore, miR-146a was necessary for the effects of elocalcitol on SCAP levels, indicating its important role in the regulation of SREBP-mediated lipogenesis.

## 4 Discussion

Our experiments demonstrate the high efficacy of vitamin D analog elocalcitol in the prevention of HFD-induced obesity and metabolic syndrome. Repeated administration of elocalcitol effectively prevented the development of almost all signs of obesity and metabolic syndrome, such as body weight gain, fat accumulation in all fat compartments, glucose intolerance, and insulin resistance in HFD-fed mice. Also, elocalcitol demonstrated preventive efficacy against liver steatosis as was evidenced by the reduction of lipid droplets accumulation in hepatocytes. Vitamin D, given at the same dose as elocalcitol demonstrated much lower efficacy. In most experiments, only a trend toward reduction of body weight as well as reduction of fat accumulation was observed. The reason for this is not clear. It is not excluded that cholecalciferol is rapidly converted into calcitriol, which binds SCAP with a much lower affinity (Kawagoe et al., 2021; Asano et al., 2017). Calcitriol binds to the VDR with a very high affinity and triggers hypercalcemia even at low doses (Smith et al., 2000; Peleg et al., 2002). In contrast, elocalcitol, a 1-α-fluor-substituted, non-hypercalcemic vitamin D derivative, has about 10 times lower efficacy for VDR (Peleg et al., 2002), and, therefore, it is reasonable to propose that the preventive action of elocalcitol on obesity is mediated via targets other than the VDR. Among other targets of elocalcitol, SCAP is the most intriguing one as it is essential for the regulation of SREBP-mediated lipogenesis. Previous studies have demonstrated that 25-hydroxyvitamin D, and fluorinated vitamin D derivatives, bind to SCAP, and formed complex undergoes rapid degradation via ubiquitination, which reduces the availability of SCAP for SREBP processing and activation (Asano et al., 2017). Our experiments demonstrated that elocalcitol administration in HFD-fed mice resulted in a reduction in SCAP levels in the liver tissue, therefore it is reasonable to propose that elocalcitol acts via the same pathway. In addition, we found that the Insig1 mRNA was also upregulated by elocalcitol in HFD-fed mice. Insig-1 also binds SCAP, resulting in the blockade of the export of SREBP to Golgi complex and reduced lipogenesis (Li et al., 2003; Takaishi et al., 2004). In transgenic mice overexpressing Insig-1 gene, the elevated Insig-1 levels inhibited SREBP processing and insulin-stimulated lipogenesis (Engelking et al., 2004). A higher Insig-1 to SCAP ratio has been shown to sequester the Insig-1/SCAP/SREBP complex in the ER, thereby reducing the transcriptional activity of SREBP (Engelking et al., 2004). While SCAP degradation by vitamin D analogs is independent of the SCAP binding to Insig-1 (Asano et al., 2017), the Insig-1 upregulation by elocalcitol in HFD-fed mice may further augment the SREBP inhibition. Currently, we do not know whether the observed upregulation of Insig1 mRNA is due to the direct action of elocalcitol or it is a compensatory reaction to the reduction of SCAP availability for Insig-1 and SREBP. Further experiments are needed to clarify this issue. By contrast vitamin D did not affect either SCAP or Insig-1 expression in HFD fed mice. Another important finding of our study is that elocalcitol administration reduced the accumulation of pro-inflammatory cytokines and induced upregulation of miR-146a. The upregulation of miR-146a was observed in both lean and obese animals. It is well documented that rapid induction of miR-146a provides a negative feedback regulation of molecules in the same signaling system used for its own induction in an effort to dampen the magnitude of the immune response. MiR-146a has been shown both *in vitro* and *in vivo* to directly target interleukin-1 receptor-associated kinase 1 (IRAK1) and tumor necrosis factor (Tnf) receptor-associated factor 6 (Traf-6), that become associated with the interleukin-1 receptor (Il-1R) upon stimulation and are partially responsible for Il-1-induced upregulation of NF-κB. This binding results in the suppression of the expression of NF-κBs target genes such as the interleukins Il-6, Il-8, Il-1β, and Tnfα [For rev see Saba et al. (2014)]. In our experiments on miR-146a-KO mice, administration of elocalcitol failed to affect the development of obesity, and these data demonstrate the importance miR-146a in the anti-obese effects of elocalcitol. Furthermore, elocalcitol failed to affect SCAP in these animals, suggesting that upregulation of miR-146a, at least in part, is responsible for the inhibitory effects of elocalcitol on the SREBP-mediated lipogenesis. It is not excluded that miR-146a regulates lipogenesis via modulation of Tnfα expression. Previous studies have shown that Tnfα is capable of inducing Srebp-1 proteolytic activation independent of the presence of sterols via neutral sphingomyelinase (Lawler et al., 1998). Additionally, it has been shown in primary porcine adipocyte cultures that miR-146a can inhibit lipogenesis via direct targeting of insulin receptors (Wu et al., 2016). The precise mechanism by which elocalcitol affects miR-146a expression remains unknown and needs further elucidation. More studies using cellular models are clearly needed to address these questions.

In our experiments, vitamin D did not demonstrate any effect on miR-146a expression and this indicates that the effects of elocalcitol on miR-146a expression occur independently of its action on VDR. Previous studies where the effects of vitamin D on the expression of miR-146a provided with controversial results. In experiments on the culture of liver stellate cells, 1α,25-dihydroxyvitamin D_3_ induced expression of miR-146a in a TGF beta-dependent manner (Zhou et al., 2017). In another study on human adipocytes, 1,25-dihydroxyvitamin D_3_ preincubation prevented Tnfα induced miR-146a expression (Karkeni et al., 2018). The discrepancy between our study and the study of Karkeni et al. (2018) is currently unknown. Probably the differences in dose of vitamin D and route of administration could account for this discrepancy.

In conclusion, our study shows that elocalcitol unambiguously prevents HFD-induced obesity and metabolic syndrome in mice. Those effects are most probably mediated via the inhibition of SREBP-mediated lipogenesis. Our data also demonstrate that the effects of elocalcitol on the obesity are related to the upregulation of miR-146a. These findings open new avenues for the preventive treatment of obesity using non-hypercalcemic analogs of vitamin D.

### 4.1 Study limitations

While this study highlights the therapeutic potential of elocalcitol in preventing obesity and associated metabolic disturbances, a key limitation is the incomplete understanding of how miR-146a mediates the observed anti-obesity effects. Although we demonstrated that elocalcitol upregulates miR-146a expression and that its deletion diminishes the therapeutic effects of elocalcitol, the precise molecular mechanisms by which miR-146a regulates SCAP levels and other targets involved in lipid metabolism and inflammation remain unclear. Furthermore, this study does not compare the efficacy and safety of elocalcitol with newer anti-obesity agents, such as GLP-1 receptor agonists (e.g., semaglutide) or dual GLP-1/GIP agonists (e.g., tirzepatide), which are widely used and administered weekly via parenteral routes. Future research should address these gaps, including head-to-head comparisons with novel therapies and investigations into elocalcitol’s effectiveness in reversing already established obesity. These efforts will provide a more comprehensive understanding of elocalcitol’s therapeutic potential and clinical relevance.

## Data Availability

The raw data supporting the conclusions of this article will be made available by the authors upon request.
