# Supplementary material for: Elocalcitol, a fluorinated vitamin D derivative, prevents high-fat diet-induced obesity via SCAP downregulation and miR-146a-associated mechanisms
Source: Front Pharmacol. 2025 Jan 17;15:1505729. doi: 10.3389/fphar.2024.1505729 (PMC11783094; doi:10.3389/fphar.2024.1505729)
Supplement: Supplementary file 1 [file DataSheet1.pdf]

Supplementary Material

1 Supplementary Tables

Supplementary Table 1.

Quantitative RT-PCR primer sequences

Gene	Primer sequence 5′-3′
Il1β	Forward: TGCCACCTTTTGACAGTGATG<br>Reverse: TGTGCTGCTGCGAGATTTGA
Tnf	Forward: GCCCACGTCGTAGCAAACC<br>Reverse: TGTCTTTGAGATCCATGCCGT
Il18	Forward: TCAAAGTGCCAGTGAACCCC<br>Reverse: GGTCACAGCCAGTCCTCTTAC
Il10	Forward: GGCGCTGTCATCGATTTCTC<br>Reverse: ATGGCCTTGTAGACACCTTGG
Il13	Forward: GACCAGACTCCCCTGTGCAACG<br>Reverse: AGGGCTACACAGAACCCGCCA
Insig1	Forward: TCACAGTGACTGAGCTTCAGCA<br>Reverse: TCATCTTCATCACACCCAGGAC
Srebp1	Forward: GGCACTAAGTGCCCTCAACCT

	Reverse: GCCACATAGATCTCTGCCAGTGT
Gapdh	Forward: GTCATATTTCTCGTGGTTCACACC<br><br>Reverse: CTGAGTATGTCGTGGAGTCTACTGG

Abbreviations: *Il1β*, interleukin-1 beta; *Tnfα*, tumor necrosis factor-alpha; *Il18*, interleukin-18; *Il10*, interleukin-10; *Il13*, interleukin-13; *Insig1, insulin-induced gene 1; Srebp1, sterol regulatory element-binding protein 1c; Gapdh*, glyceraldehyde-3-phosphate dehydrogenase.

Supplementary Table 2.

Calcium levels (mmol/L) in blood serum and urine of mice fed with HFD and treated with vitamin D or elocalcitol.

Number of animals: n = 7-8.

	Experimental groups			
	Control	HFD	HFD+vitD	HFD+Eloc
Serum	2,14±0,08	1,93±0,04	2,04±0,03	1,95±0,06
Urine	1,26±0,25	1,42±0,18	1,90±0,12	1,54±0,13

Supplementary Table 3.

Calcium levels (mmol/L) in blood serum of wild-type (WT) and miR-146a knockout (KO) mice fed with HFD and treated with elocalcitol. Number of animals: n = 7-8.

	Experimental groups							
	WT Control	WT Control+Eloc	WT HFD	WT HFD+Eloc	KO Control	KO Control+Eloc	KO HFD	KO HFD+Eloc
Serum	1,99±0,04	2,03±0,12	2,35±0,14	2,36±0,05	1,98±0,10	2,10±0,04	2,31±0,08	2,28±0,07

2. Supplementary Figures

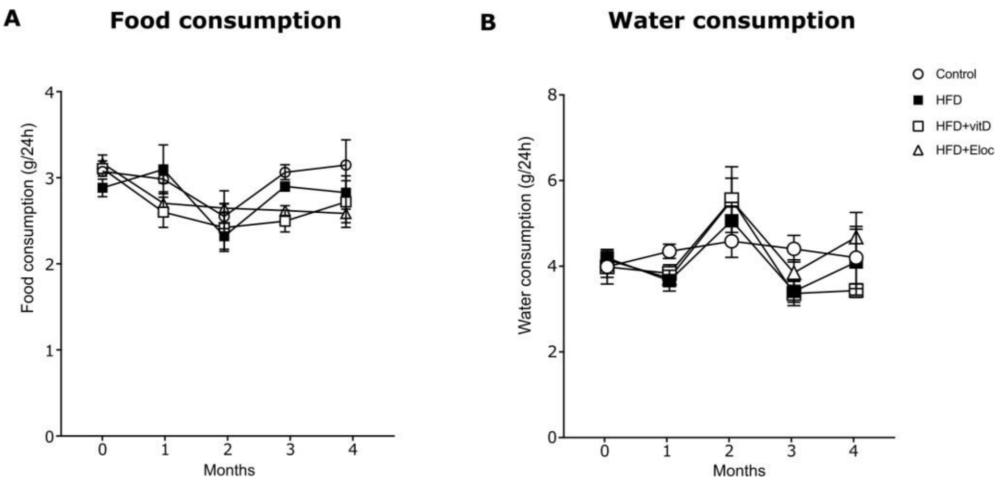

Supplementary Figure 1. The effect of vitamin D and elocalcitol on the food- (A) and water intake of mice fed with HFD and treated with vitamin D or elocalcitol.; Number of animals: n = 10.

Measurements were taken at the end of months 0, 1, 2, 3, and 4. For each time point, food and water consumption was calculated over a 24-hour period for each cage, with each cage containing 5 animals.

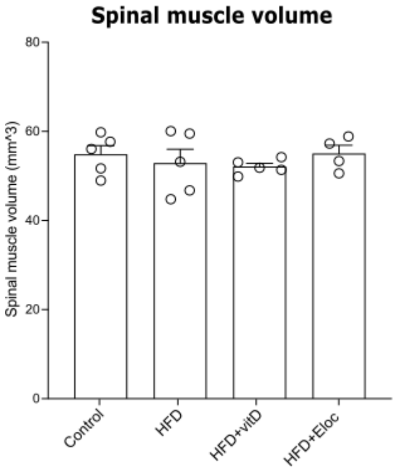

Supplementary Figure 2. The effect of vitamin D and elocalcitol on the volume of spinal muscles in mice fed with HFD and treated with vitamin D or elocalcitol. Number of animals: n = 4-5.

Volumes of spinal muscles were measured from the same MRI scans used for body composition analysis. Volumes were segmented manually by an observer blinded to the experiment using ITK-SNAP (V3.8.0). A 40x magnification was employed to assess the volumes in cubic millimeters (mm^3) of spinal muscle, with the renal region serving as the reference point for volume analysis.

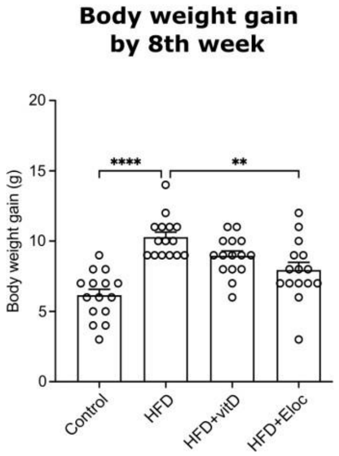

Supplementary Figure 3. The effect of vitamin D and elocalcitol on the body weight gain by 8th week of treatment. ** $p < 0.01$; **** $p < 0.0001$; One-way ANOVA followed by Tukey's multiple comparisons post hoc test. Number of animals n =15. The data are expressed as mean ± SEM.

Animals' body weights were monitored weekly throughout the study. Body weight gain by the 8th week was calculated as the difference between the body weight at week 8 and the initial body weight at the start of the experiment.
